# Amyotrophic Lateral Sclerosis Type 20 - *In Silico* Analysis and Molecular Dynamics Simulation of hnRNPA1

**DOI:** 10.1371/journal.pone.0158939

**Published:** 2016-07-14

**Authors:** Bruna Baumgarten Krebs, Joelma Freire De Mesquita

**Affiliations:** Laboratory of Bioinformatics and Computational Biology, Department of Genetics and Molecular Biology, Federal University of Rio de Janeiro State (UNIRIO), Rio de Janeiro, Brazil; Department of Pathology, Anatomy & Cell Biology, Thomas Jefferson University, UNITED STATES

## Abstract

Amyotrophic Lateral Sclerosis (ALS) is a fatal neurodegenerative disease that affects the upper and lower motor neurons. 5–10% of cases are genetically inherited, including ALS type 20, which is caused by mutations in the hnRNPA1 gene. The goals of this work are to analyze the effects of non-synonymous single nucleotide polymorphisms (nsSNPs) on hnRNPA1 protein function, to model the complete tridimensional structure of the protein using computational methods and to assess structural and functional differences between the wild type and its variants through Molecular Dynamics simulations. nsSNP, PhD-SNP, Polyphen2, SIFT, SNAP, SNPs&GO, SNPeffect and PROVEAN were used to predict the functional effects of nsSNPs. *Ab initio* modeling of hnRNPA1 was made using Rosetta and refined using KoBaMIN. The structure was validated by PROCHECK, Rampage, ERRAT, Verify3D, ProSA and Qmean. TM-align was used for the structural alignment. FoldIndex, DICHOT, ELM, D2P2, Disopred and DisEMBL were used to predict disordered regions within the protein. Amino acid conservation analysis was assessed by Consurf, and the molecular dynamics simulations were performed using GROMACS. Mutations D314V and D314N were predicted to increase amyloid propensity, and predicted as deleterious by at least three algorithms, while mutation N73S was predicted as neutral by all the algorithms. D314N and D314V occur in a highly conserved amino acid. The Molecular Dynamics results indicate that all mutations increase protein stability when compared to the wild type. Mutants D314N and N319S showed higher overall dimensions and accessible surface when compared to the wild type. The flexibility level of the C-terminal residues of hnRNPA1 is affected by all mutations, which may affect protein function, especially regarding the protein ability to interact with other proteins.

## Introduction

Amyotrophic Lateral Sclerosis (ALS) is a neurodegenerative disease that affects the upper and lower motor neurons, causing weakness, muscle atrophy, and eventually death [1]. ALS is one of the most frequent types of motor neuron diseases, with an incidence of 1–5 per 100,000, and thus, it is extensively studied [2]. The ALS onset age is usually around age 40, being juvenile ALS rare [1]. Due to the lack of an effective treatment, ALS leads to death between 2 and 5 years after diagnosis, mostly due to respiratory failure [2]. Although most ALS cases are sporadic (sALS), 5–10% are familial (fALS) and related with inherited genetic mutations. Among the previously identified ALS causative genes, the most frequently mutated ones are C9orf72, SOD1, TARDBP and FUS [3].

**Fig 1:**
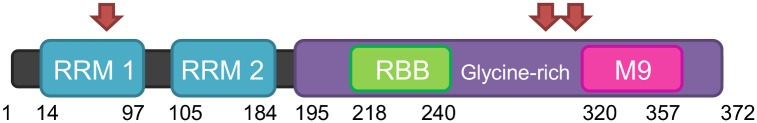
Schematic representation of the domains found on hnRNPA1. The two RNA recognition motifs (RRM 1 and 2) are represented in blue, the glycine-rich domain is represented in purple, the RNA-binding box is represented in green, and the nuclear localization signal M9 is represented in pink. The red arrows indicate the location where the four known mutations occur: position 73 (mutation N73S), position 314 (mutations D314V and D314N) and position 319 (mutation N319S).

Recently, mutations in the hnRNPA1 gene were identified in one family with ALS and in one sporadic ALS case [4]. The hnRNPA1 gene codes for the ROA1 protein, usually referred to as hnRNPA1 as well. This heterogeneous nuclear ribonucleoprotein (hnRNP) plays a key role in mRNA metabolism, being involved in alternative splicing, nucleocytoplasmic shuttling and microRNA biogenesis [5–7]. Along with histones, hnRNPs are the most abundant proteins in the nucleus [8]. Two RNA recognition motifs, one RNA-binding box, one M9 nuclear localization signal, and a príon-like glycine-rich domain in the C-terminal part of the protein have been previously identified in hnRNPA1 [8]; however, its complete tridimensional structure has not yet been experimentally solved (Fig 1).

The knowledge of tridimensional structures allows for a better understanding of the activity of a protein, the structure-function relationship, the interaction with other molecules, and contributes for a better comprehension of biological processes in a more detailed approach. With the advances in sequencing technology, the number of protein sequences available in online databases has grown exponentially, producing an extensive amount of data. The conventional methods of protein structure determination, such as crystallography, electron microscopy or nuclear magnetic resonance (NMR), are time consuming and expensive [9]. In this scenario, the computational approach of Bioinformatics comes as a great ally of experimental methodology. Computational—or *in silico—*methods are based on algorithms that can make predictions with a variety of purposes, such as predicting the effect of mutations in protein function according to the amino acid sequence, and modeling tridimensional structures in a cheaper, faster, and yet efficient way.

In this work, computational biology methods were applied, following the methodology previously described by our group [10,11], to an *in silico* analysis of hnRNPA1 protein, which has been described as the cause of familial Amyotrophic Lateral Sclerosis type 20, aiming for a thorough analysis of the protein structure and its natural variants, as well as the effects of structural changes in the disease development.

## Materials and Methods

### Sequence Retrieval

The sequence of hnRNPA1 and its natural variants were retrieved from the UNIPROT database [UniProt ID: P09651] and OMIM [OMIM ID: 164017].

### SNP Analysis

Eight algorithms were used to analyze the functional effects of non-synonymous single nucleotide polymorphisms: nsSNP [12], PhD-SNP [13], Polyphen2 [14], SIFT [15], SNAP [16], SNPs&GO [17], SNP Effect [18] and PROVEAN [19].

### Structural Modeling

The tridimensional structures were created based on comparative and *ab initio* modeling. For the comparative modeling, the following algorithms were used: IntFOLD [20], Phyre2 [21], M4T [22], SwissModel [23], PS2 [24], RaptorX [25] and Modeller [26]. For the *ab initio* modeling, the algorithms Rosetta [27,28] and I-TASSER [29] were used. The generated structures were then structurally aligned to the crystallographic structure of hnRNPA1 (PDB ID: 1L3K), which comprises its first 196 amino acids, using the TM-Align server [30], and the best structures were chosen according to the RMSD and TM-score values.

### Structure Refinement

The selected structures were submitted to KoBaMIN, a structure refinement algorithm that performs stereochemistry correction, and energy minimization using a knowledge-based potential of mean force [31].

### Structure Validation

The selected structures had their quality analyzed through the following structure validation algorithms: PROCHECK [32], Rampage [33], Qmean server [34], ProSA web [35], ERRAT [36] and Verify3D [37]. To further validate the modeled structure, its secondary structure was predicted by PsiPred [38], JuFo9D [39] and Jpred [40], and six disorder prediction algorithms were also consulted: FoldIndex [41], Disopred [42], ELM [43], DisEMBL [44], DICHOT [45] and D2P2 [46].

### Conservation Analysis

The phylogenetic analysis was performed using the ConSurf algorithm [47,48], which determined the evolutionary conservation degree of each hnRNPA1 amino acid. The analysis was done using UniProt database, with a maximum of 95% of identity between sequences, and a minimum of 35% of identity for homologs.

### Molecular Dynamics

The GROMACS package version 5.0.7 [49] was used for the molecular dynamics (MD) simulations of the wild type structure and the natural variants D314N, D314V, N73S and N319S. The tridimensional structures of the natural variants were generated using the Mutator plugin available in the VMD software (Version 1.9.2) [50]. The force field used was Amber99SB-ILDN [51]. The molecules were solvated in a dodecahedral box with TIP3P water molecules, and neutralized by adding Na^+^Cl^-^ ions. The energy minimization was carried out using steepest descent method for 5000 steps. After minimization, NVT (constant number, volume and temperature) equilibration was done, with constant temperature of 300K for 100ps, followed by NPT (constant number, pressure and temperature) equilibration, with constant pressure of 1 atm and constant temperature of 300K for 100ps. The production simulations were performed at 300K for 40ns. The algorithm LINCS (Linear Constraint Solver) was used to constrain the covalent bonds [52], and the electrostatic interactions were computed using the Particle Mesh Ewald (PME) method [53]. The MD trajectories were saved every 10ps. The stability and conformational changes in the native and the mutants were assessed through the analysis of Root-mean-square deviation (RMSD), Root-mean-square fluctuation (RMSF), Radius of gyration (Rg), Number of hydrogen bonds (Hb), Solvent accessible surface area (SASA), and B-factor. All graphs were created using the XMGrace tool [54].

## Results and Discussion

### Sequence and natural variants retrieval

HnRNPA1 is a 372 amino acid protein (isoform A1-B) coded by the hnRNPA1 gene, which is located on chromosome 12q13.13. There are four natural variants currently known: N73S, D314V, D314N and N319S (Fig 1).

The D314N and N319S mutations were identified in patients with Amyotrophic Lateral Sclerosis type 20, the first one in a family, and the second one in a patient with sporadic ALS, while the D314V mutation was identified in a family with inclusion body myopathy and Paget’s disease of the bone [4]. The N73S mutation has not been correlated with any diseases so far.

### nsSNP Analysis

**Fig 2:**
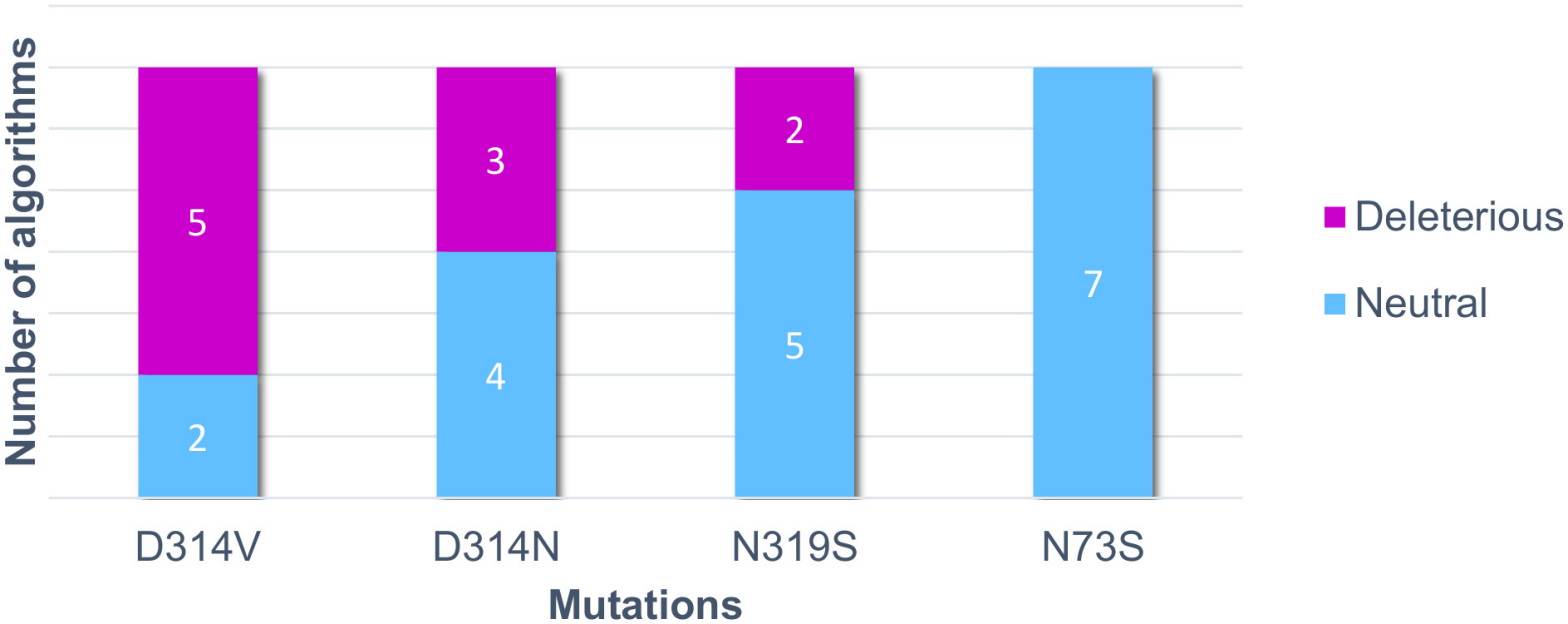
Number of “deleterious” and “neutral” predictions of each hnRNPA1 mutation. The four known mutations were analyzed by non-synonymous single nucleotide polymorphism (nsSNP) prediction algorithms. The graph indicates how many algorithms predicted each mutation as having a deleterious effect or a neutral effect on hnRNPA1. Blue bars indicate neutral predictions, and purple bars indicate deleterious predictions.

The natural variants were functionally analyzed by different algorithms that predict whether they have deleterious or neutral effect on protein function. The N73S mutation was the only one predicted as neutral by all the algorithms, while the D314V, D314N and N319S mutations were predicted as deleterious by at least two algorithms (Fig 2). The D314V mutation was predicted as deleterious by PhD-SNP, Polyphen-2, SIFT, SNAP and PROVEAN. The algorithms SIFT, SNAP and PROVEAN predicted the D314N variant as being deleterious, and the N319S variant was predicted as deleterious by PhD-SNP and SIFT (Table 1).

**Table 1:** Functional effect prediction of hnRNPA1 natural variants by different SNP prediction algorithms.

	Non-synonymous SNP analysis algorithms						
Natural variant	nsSNP Analyzer	PhD-SNP	Polyphen-2	SIFT	SNAP	SNPs&GO	PROVEAN
N73S	Neutral	Neutral	Neutral	Neutral	Neutral	Neutral	Neutral
D314V	Neutral	Deleterious	Deleterious	Deleterious	Deleterious	Neutral	Deleterious
D314N	Neutral	Neutral	Neutral	Deleterious	Deleterious	Neutral	Deleterious
N319S	Neutral	Deleterious	Neutral	Deleterious	Neutral	Neutral	Neutral

The inconsistency between results shows how important it is to use more than one prediction algorithm to determine the potential effects of mutations. While most algorithms successfully predicted the D314V mutation as deleterious, 4 out of 7 algorithms failed to suggest the D314N variant’s deleterious potential, as well as 5 out of 7 algorithms failed to predict the N319S variant as deleterious, suggesting that the results obtained with the nsSNP prediction algorithms are not conclusive. The variants were further analyzed using SNP Effect, which predicts the mutations effect on aggregation tendency (TANGO), amyloid propensity (WALTZ) and chaperone binding tendency (LIMBO) (Table 2). SNP Effect results showed that aggregation tendency and chaperone binding tendency are not affected by any variant, but the D314N mutation increases amyloid propensity, while N319S decreases amyloid propensity. Mutation D314V was shown to increase amyloid propensity, corroborating the experimental findings by Shorter and Taylor [55].

**Table 2:** SNP Effect predictions on hnRNPA1 natural variants.

	SNP Effect		
Natural variant	TANGO (Aggregation tendency)	WALTZ (Amyloid propensity)	LIMBO (Chaperone binding tendency)
N73S	Unaffected	Unaffected	Unaffected
D314V	Unaffected	Increase	Unaffected
D314N	Unaffected	Increase	Unaffected
N319S	Unaffected	Decrease	Unaffected

### Structural Modeling

Comparative modeling is a technique that builds tridimensional protein models based on experimentally determined structures of homologous proteins [56]. HnRNPA1 has had part of its structure previously defined experimentally, corresponding to the first 196 amino acids [PBD ID: 1L3K], which comprises the two RNA-recognition motifs, but not the prion-like glycine-rich domain or the M9 nuclear targeting sequence. This structure was therefore used as the template for the comparative modeling, which was performed using different algorithms: IntFOLD, Phyre 2, M4T, SwissModel, PS2, RaptorX and Modeller.

*Ab initio* modeling predicts protein structure based on thermodynamics concepts, assuming that all the information needed is within the amino acid sequence, and that the native structure of a protein corresponds to the global minimum of its free energy [9,57]. I-TASSER and Rosetta, the two algorithms considered as the most successful predictors according to CASP (Critical Assessment of protein Structure Predicion) experiments [9], were used for the *ab initio* modeling.

The generated models were compared to the 1L3K fragment using the structural alignment program TM-align. The alignment provides two distinct values: Root-mean-square deviation (RMSD), which measures the distance between corresponding residues, and TM-score, a structural similarity measure that balances RMSD and coverage [58]. Accurate models present RMSD < 2.0Å, and TM-scores tending to 1. The values of the structural alignment are summarized on Table 3.

**Table 3:** RMSD and TM-score values after structural alignment between modeled structures and the solved fragment (PBD ID: 1L3K) using TM-align.

	RMSD (Å)	TM-score
**IntFOLD (model 2)**	0.61	0.98420
**PS2**	0.26	0.99698
**Phyre2**	2.24	0.83087
**M4T**	0.42	0.99249
**Swiss-Model (model 2)**	0.68	0.98513
**RaptorX**	0.92	0.96971
**Modeller**	0.64	0.98271
**I-TASSER (model 4)**	0.35	0.99527
**Rosetta (model 2)**	0.50	0.98918

Most algorithms generated high quality models according to their RMSD and TM-score values. However, most comparative modeling programs failed to model the C-terminal part of the protein. Even though PS2 had the best scores, the generated structure was not visually accurate, since the C-terminal part of the protein was not modeled. The same issue was seen in the models created by IntFOLD, M4T, Swiss-Model, Raptor X and Modeller. The structures modeled by I-TASSER, Rosetta and Phyre 2 successfully modeled the C-terminal portion, however, since Phyre 2 structure showed an RMSD higher than 2.0 Å, it was not considered. Although I-TASSER and Rosetta generated five structures each, only Rosetta model 2 and I-TASSER model 4 scored an RMSD value under 2.0 Å, suggesting that those are reliable models (Table 3). Both structures were submitted to the refinement algorithm KoBaMIN, which performed energy minimization and stereochemistry correction.

### Structure Validation

**Fig 3:**
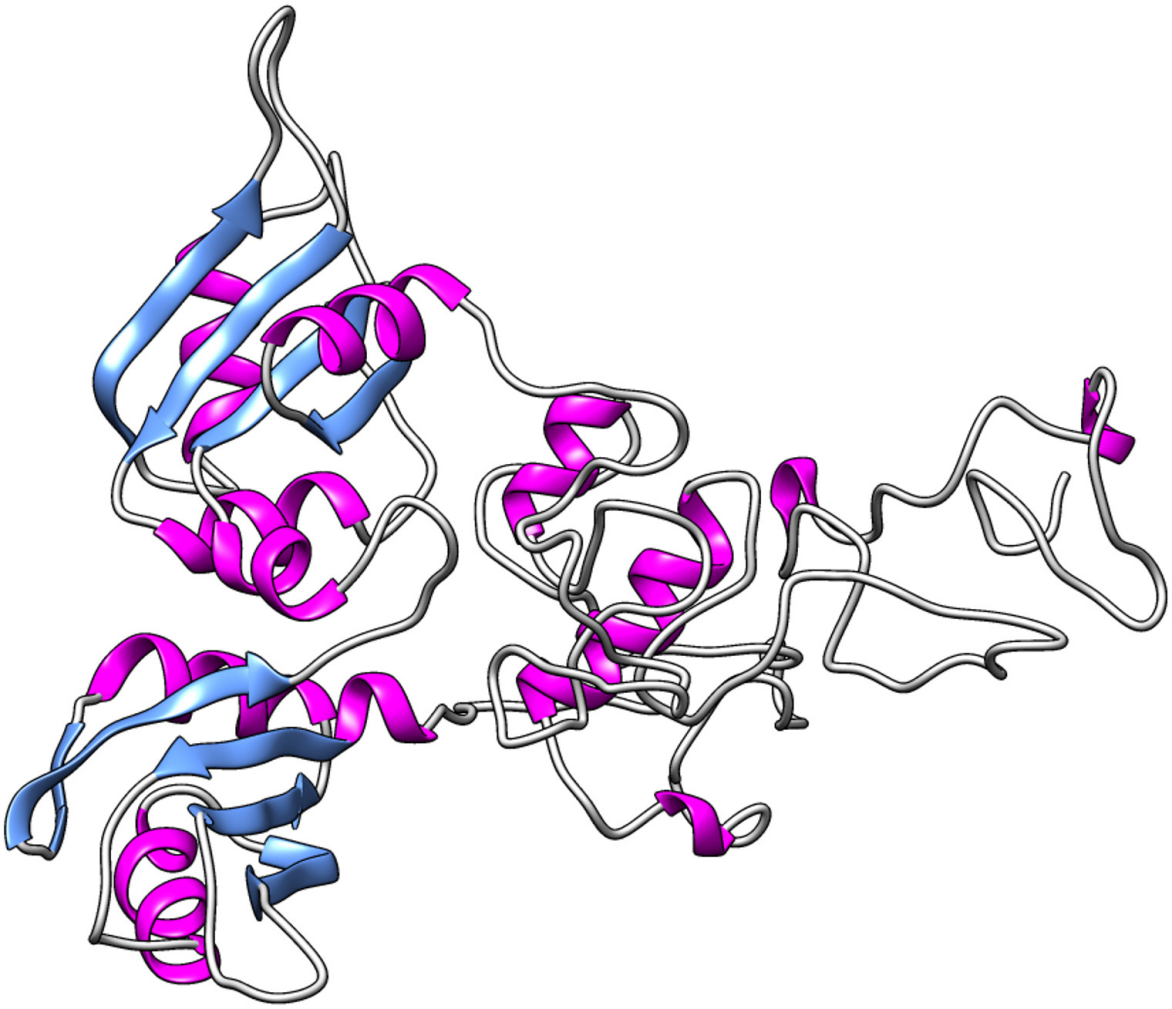
*In silico* modeled structure of hnRNPA1. The tridimensional model of hnRNPA1 generated using Rosetta algorithm followed by refinement using KoBaMIN. The α-helix are represented by pink ribbons, the β-strands are represented by blue arrows, and the coiled regions are represented in grey.

The two refined structures were assessed by the validation algorithms PROCHECK, Rampage, Verify3D, ERRAT, ProSA and Qmean to determine which structure should be used for the next steps. The structure modeled by Rosetta, followed by refinement by KoBaMIN, obtained better scores in all algorithms when compared to the structure modeled by I-TASSER, and therefore was selected as the final model (Fig 3).

**Fig 4:**
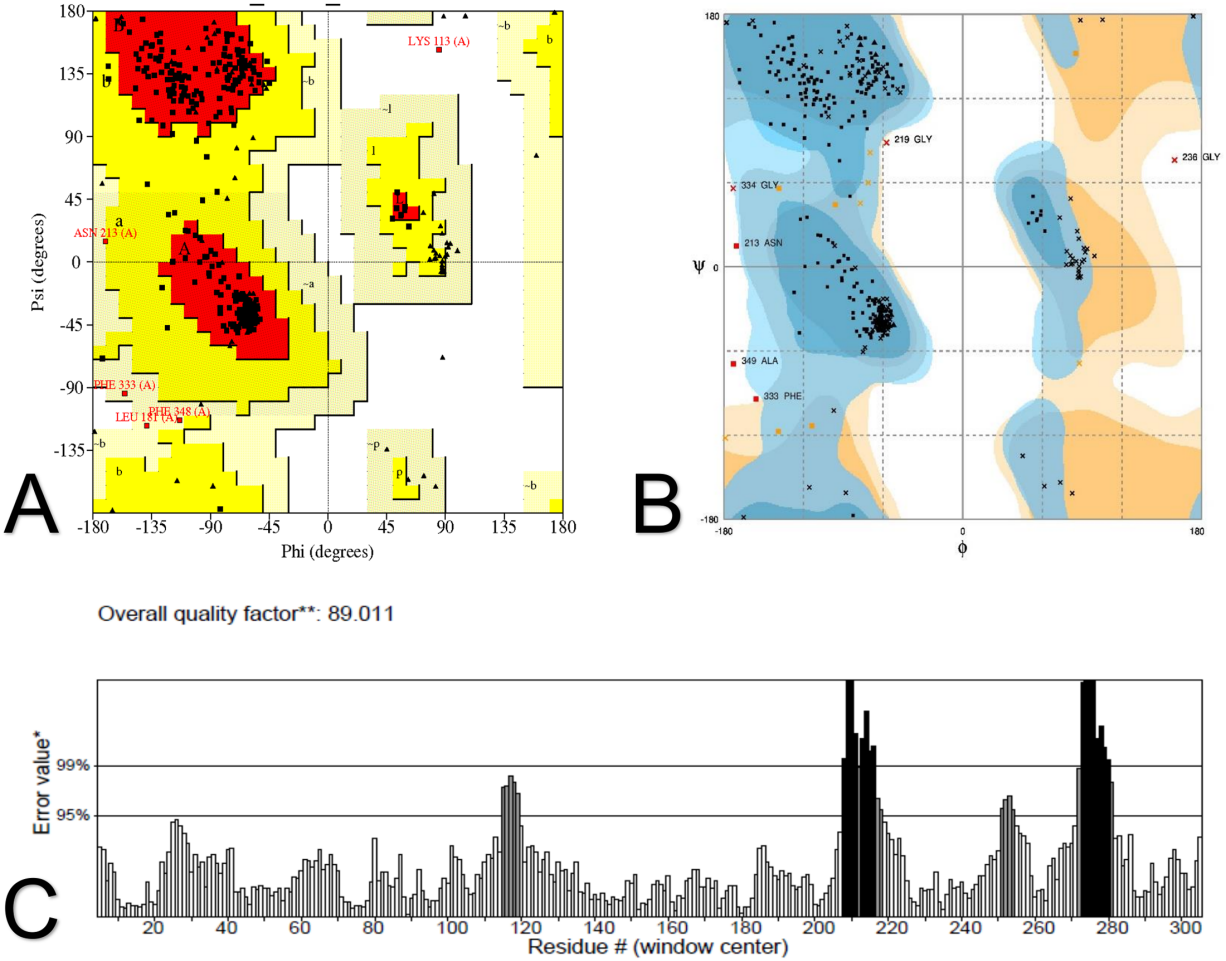
Validation of the *in silico* modeled hnRNPA1 structure. The modeled structure was validated by PROCHECK, Rampage and ERRAT. (A) PROCHECK’s Ramachandran plot indicates that 89.9% of residues lie in most favored regions, 8.2% in additional allowed regions, 1.5% in generously allowed regions and 0.4% in disallowed regions. (B) Rampage’s Ramachandran plot shows 95.7% of residues in favored regions, 2.7% in allowed regions and 1.6% in outlier regions. (C) According to ERRAT, the structure obtained an 89.011 overall quality factor.

Ramachandran plots were obtained from PROCHECK and Rampage, which are algorithms that check the overall stereochemical quality of a protein structure. The plots show the phi(Φ)-psi(ψ) torsion angles for every residue of a protein. The final model had 89.9% residues lying in most favored regions, 8.2% in additional allowed regions, 1.5% in generously allowed regions and 0.4% in disallowed regions on the plot generated by PROCHECK (Fig 4A). On the Ramachandran plot generated by Rampage, the final model had 95.7% residues in favored regions, 2.7% in allowed regions and 1.6% in outlier regions (Fig 4B). Although excellent quality models are expected to have over 90% residues in the most favored regions of PROCHECK’s Ramachandran plot, and around 98% in the favored regions of Rampage’s Ramachandran plot, the final model was considered of good quality since it scored 89.9% and 95.7% respectively.

The good quality of the final model was confirmed by Verify3D, an algorithm that analyzes the accuracy of the tridimensional model by comparing it to its own one dimensional amino acid sequence, since 99.46% of the residues showed a 3D-1D score higher than 0.2. The ERRAT plot for the final model exhibited an 89.011 overall quality factor, further supporting its good quality as scores higher than 50 are considered acceptable (Fig 4C) [36,59].

**Fig 5:**
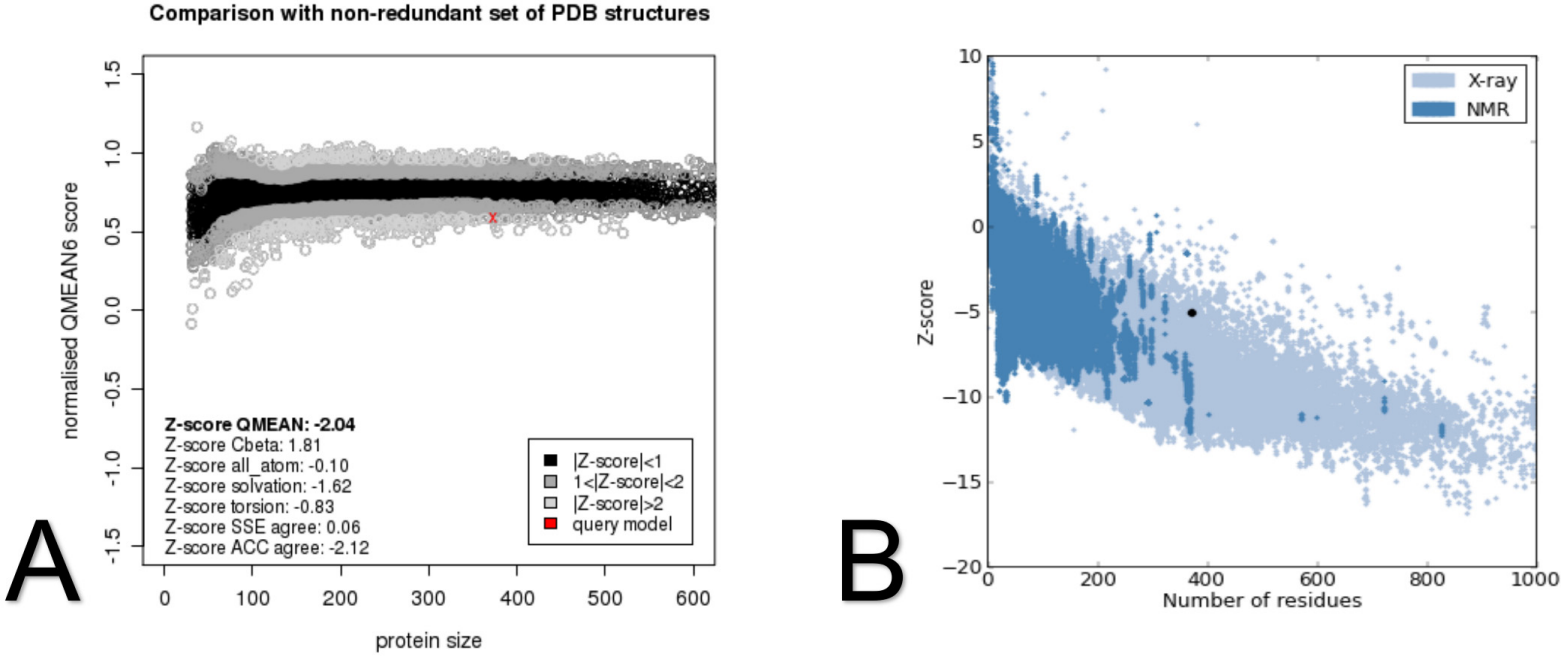
Validation of the *in silico* modeled hnRNPA1 structure by Qmean and ProSA web. (A) Structure validation by Qmean, which shows the quality of a structure when compared to a non-redundant set of PDB structures of the same size. The image shows that the modeled structure’s score, indicated by the red “X”, falls within the range of scores of reference structures of the same size, and it is therefore of good quality. (B) Structure validation by ProSA web algorithm. The graph shows that the modeled structure’s score, indicated by the black dot, falls within the range of scores found on similar sized proteins, with an X-ray quality.

The QMEAN Z-score measures the absolute quality of a model by comparing its QMEAN score to scores of experimentally solved proteins. The final model obtained a QMEAN score of 0.593 and a QMEAN Z-score of -2.04, which falls within the range of scores found for reference structures of the same size (Fig 5A). A similar analysis is made by ProSA web algorithm, according to which the final structure obtained a Z-score of -5.05 and therefore also falls within the range of scores found on similar sized proteins, with an X-ray quality (Fig 5B).

To further validate the modeled structure, we carried an in depth analysis of the C-terminal end of hnRNPA1, inspecting if the disordered aspect of the final model was reliable. The C-terminal part of hnRNPA1 consists of a glycine-rich region that harbors a nuclear targeting sequence (M9), responsible for the shuttling between the nucleus and the cytosol, and an RNA-binding motif [8]. The C-terminal end of hnRNPA1 is also known to be essential for its activity, mediating protein-protein interactions [4]. Although it has not been experimentally shown, previous studies suggest that the C-terminal end of hnRNPA1 is intrinsically unfolded [4,55]. The amino acid sequence was submitted to three secondary structure prediction algorithms (Jufo, Jpred and PsiPred), and to six intrinsically disordered protein prediction algorithms (FoldIndex, Disopred, ELM, DisEMBL, DICHOT and D^2^P^2^).

All three secondary structure prediction algorithms agreed that the predominant secondary structure on the C-terminal half of hnRNPA1 is coil, except for amino acids number 229, 347, 370, 371 and 372, which were predicted as coil by PsiPred and Jpred, but predicted as strand by JuFo. The results obtained by the six predictors of intrinsic protein disorder suggest that the C-terminal half of hnRNPA1 is indeed intrinsically disordered. ELM, D^2^P^2^ and DisoPred considered that most amino acids from the C-terminal part are disordered, while FoldIndex as well as DICHOT and DisEMBL predicted the entire C-terminal part as intrinsically disordered. With the confirmation of all cited algorithms, the modeled structure was considered of good quality and was therefore used for the next steps.

### Molecular Dynamics

To further analyze the effects of mutations in hnRNPA1, we performed MD simulations using the software GROMACS 5.0.7, and compared the simulation results between the wild type and its natural variants. The tridimensional structure modeled in this work was used as the wild type structure, and the structures of the four known mutations were generated using the Mutator plugin available in the VMD software.

MD simulations aim to reproduce the real behavior of molecules in their environment, taking in consideration its flexibility and movement, rather than the static picture obtained from methods such as crystallography [60]. These simulations can provide detailed information regarding particle motions as a function of time [61]. The MD simulations were performed for 40ns to investigate stability and conformational changes on hnRNPA1 structure upon mutation. The analyzed parameters were RMSD, RMSF, Rg, SASA and B-factor.

**Fig 6:**
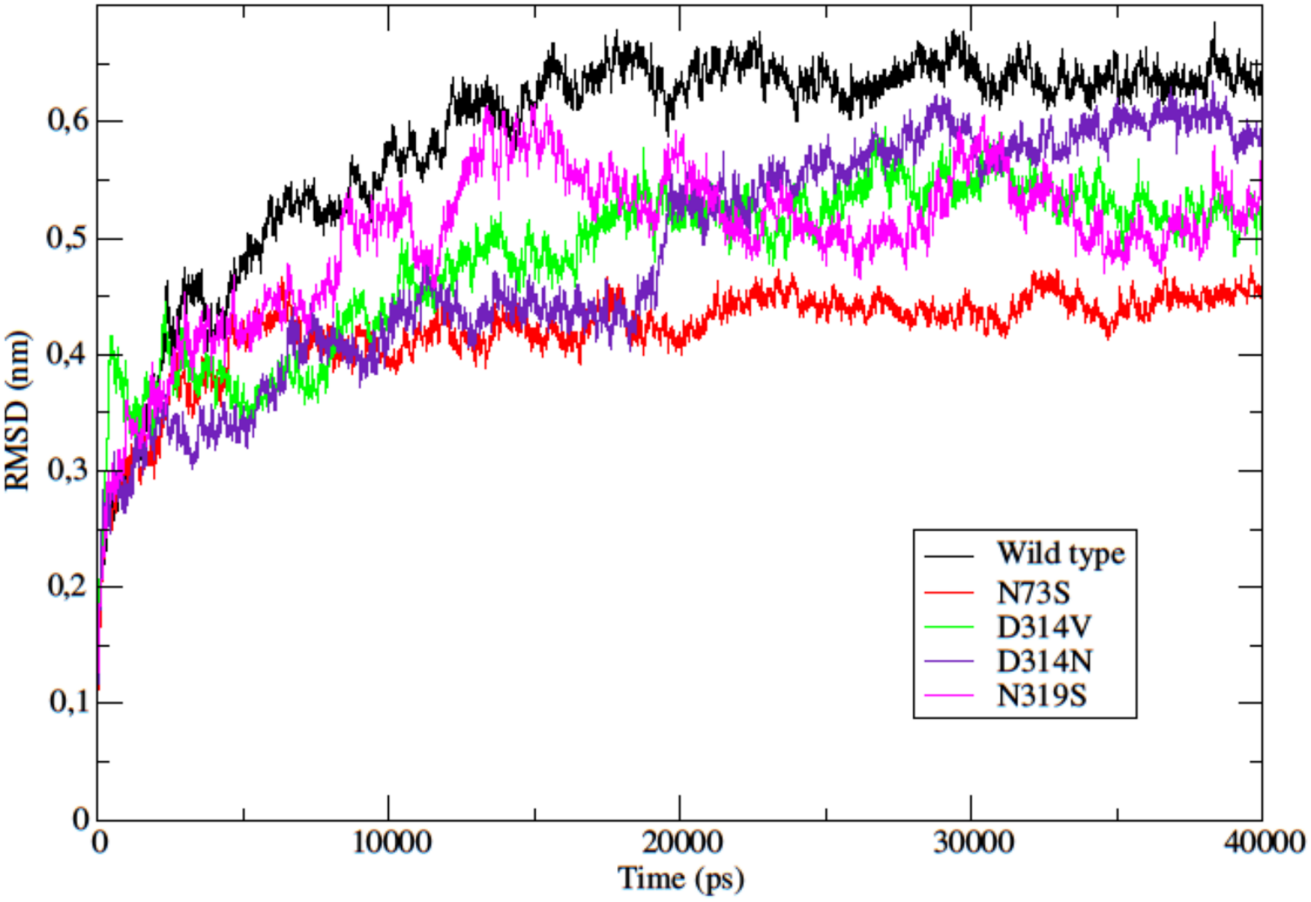
Backbone RMSD as a function of time. The RMSD for the backbone atoms of the wild type and the mutants are shown as a function of time. Wild type is represented in black, mutant N73S in red, mutant D314V in green, mutant D314N in purple, and mutant N319S in pink.

The RMSD of the backbone atoms is a useful parameter to assess the equilibration and stabilization of MD trajectories. As shown in Fig 6, the RMSD for the backbone atoms of the wild type protein stabilized at 0.65nm after 15ns, and did not show any significant changes after that, which suggests that the modeled protein is stable throughout the simulation. The average RMSD of all structures ranged from 0.2 to 0.7nm during the trajectory. The N73S mutant stabilized after the first 10ns, with an average RMSD of 0.45nm, and kept constant until the end of the MD simulation. The D314V mutant stabilized around 20ns with an average RMSD of 0.55nm. The D314N mutant seemed to stabilize with an RMSD of 0.45nm, but at 20ns it showed an increase in the backbone RMSD to 0.6nm. To confirm that this mutant’s trajectory stayed stable with an RMSD of 0.6nm, we carried out a longer MD simulation of 100ns. This longer trajectory showed that this mutant actually stays stable and does not show other abrupt increases or decreases in the RMSD value (S1 Fig). The N319S mutant seemed to stabilize around 20ns with an average RMSD of 0.5nm. This mutant’s trajectory showed an increase in the backbone RMSD at 30ns to 0.6nm. The RMSD analysis of the wild type and the mutants shows that all mutations affect the protein stability, and that all four mutations result in an increase in stability of hnRNPA1.

**Fig 7:**
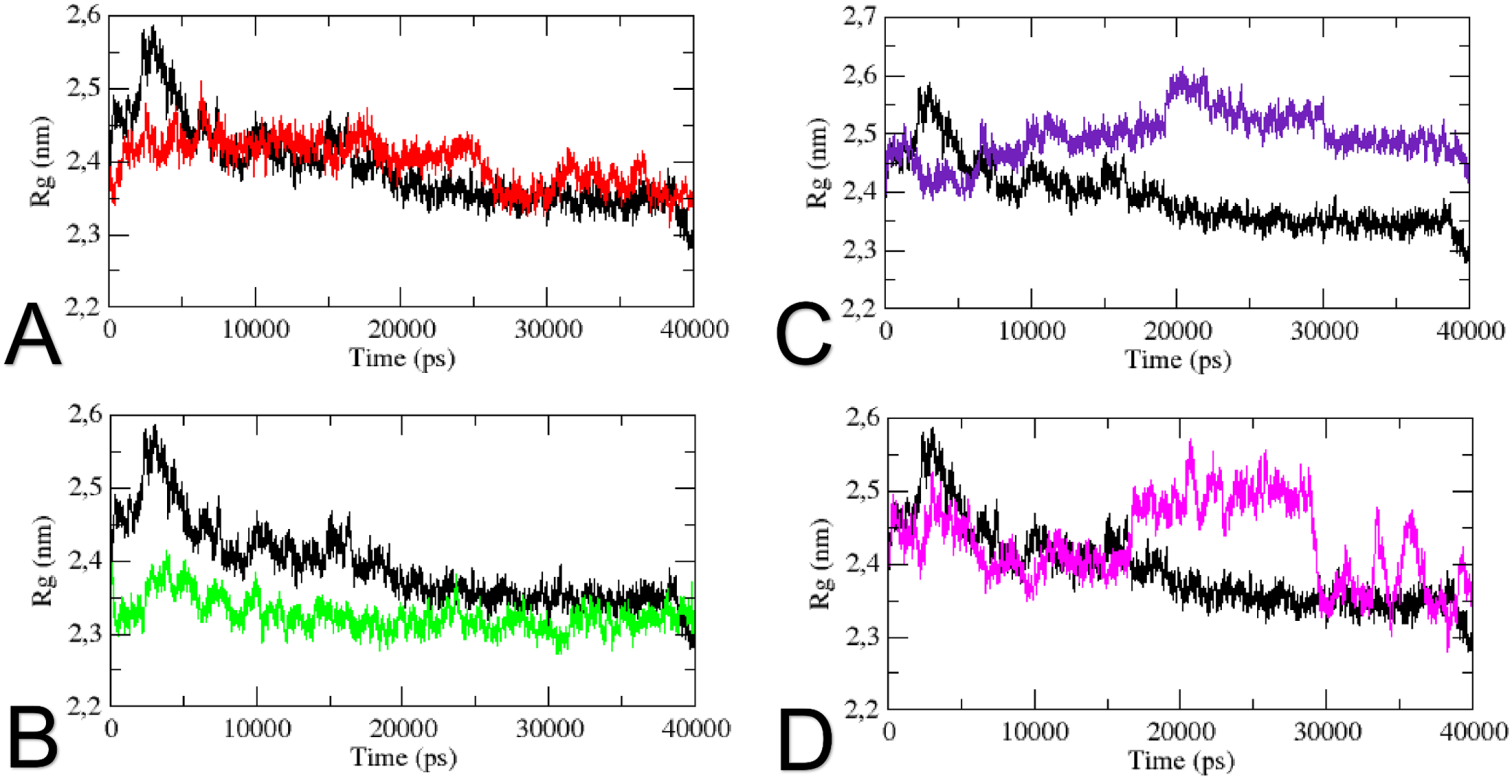
Radius of gyration (Rg) of Cα atoms as a function of time. The Radius of gyration of Cα atoms of the wild type and the mutants during the MD trajectory is shown. (A) The wild type is represented in black, and mutant N73S in red. (B) The wild type is represented in black, and mutant D314V in green. (C) The wild type is represented in black, and mutant D314N in purple. (D) The wild type is represented in black, and mutant N319S in pink.

The radius of gyration analysis indicates the level of compaction of each molecule [62] and the overall dimensions of the structure [63]. As shown in Fig 7, the wild type structure shows an average Rg value of 2.35nm after 20ns, with a slight decrease at the end of the simulation. All mutants showed to affect the level of compaction of hnRNPA1. The Rg value of the N73S mutation follows the same pattern as the wild type protein after 10ns, except for a slight increase in the Rg value to 2.45nm at 25ns, where the wild type protein Rg is about 2.32nm. The D314V mutant shows a constant Rg value of 2.35nm throughout the simulation, presenting a greater level of compaction when compared to the wild type structure. Mutant D314N shows an average Rg of 2.5nm after 10ns, and an increase is seen at 20ns, when the Rg value rises to 2.6nm. This result suggests that this mutation causes the protein to increase its overall dimension. The Rg value of the N319S mutant seems to follow the same pattern as the wild type up until 15ns, where the Rg value shows a significant increase of 0.25nm, reaching an Rg of 2.58nm. A significant decline is seen around 28ns when the Rg value decreases from 2.55nm to 2.3nm, and two similar fluctuations are seen until the end of the trajectory. This analysis suggests that, except for mutant N73S, all mutations significantly affect the protein level of compaction.

**Fig 8:**
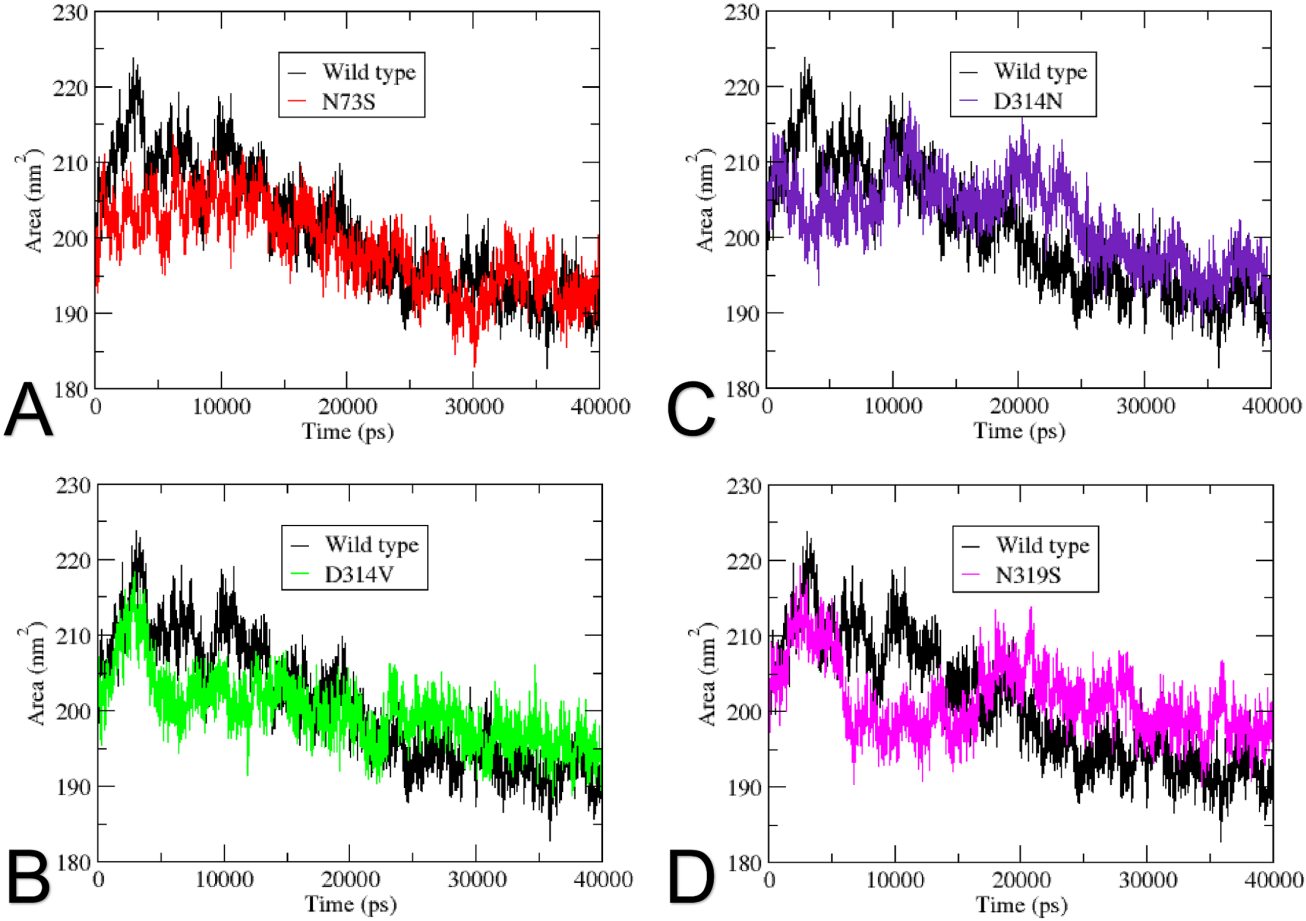
Solvent-accessible surface area (SASA) as a function of time. SASA of the wild type and the mutants is shown as a function of time. (A) The wild type is represented in black, and mutant N73S in red. (B) The wild type is represented in black, and mutant D314V in green. (C) The wild type is represented in black, and mutant D314N in purple. (D) The wild type is represented in black, and mutant N319S in pink.

The SASA analysis assesses the surface area of the protein that is accessible to the solvent [63], where high SASA values suggest relative expansion. As seen in Fig 8, the wild type protein starts with a SASA value of 210nm^2^, reaches its highest SASA value of 224nm^2^ at 300ps, and gradually decreases to 186nm^2^ at the end of the trajectory. Mutant N73S obtained an overall similar pattern when compared to the wild type protein, gradually decreasing throughout the simulation, without significant differences in SASA values. The D314V mutant presents a dynamic plot, starting with lower SASA values when compared to the wild type in the first 23ns, and increasing its SASA values until the end of the trajectory. This mutant achieves its highest SASA value of 217nm^2^ at 300ps. Mutant D314N starts with SASA values lower than the wild type, but an increase can be seen at 10ns, when the mutant achieves higher SASA values when compared to the wild type protein. This increase in SASA value suggests that this mutant is relatively more accessible than the wild type protein. Mutant N319S presents a similar SASA pattern as mutant D314N. It starts the trajectory with lower SASA values as compared to the wild type, but an increase can be seen at 18ns, when the mutant achieves SASA values of 215nm^2^, keeping higher values than the wild type until the end of the trajectory. High SASA values indicates an increase in the accessible surface of the mutant relatively to the wild type protein.

**Fig 9:**
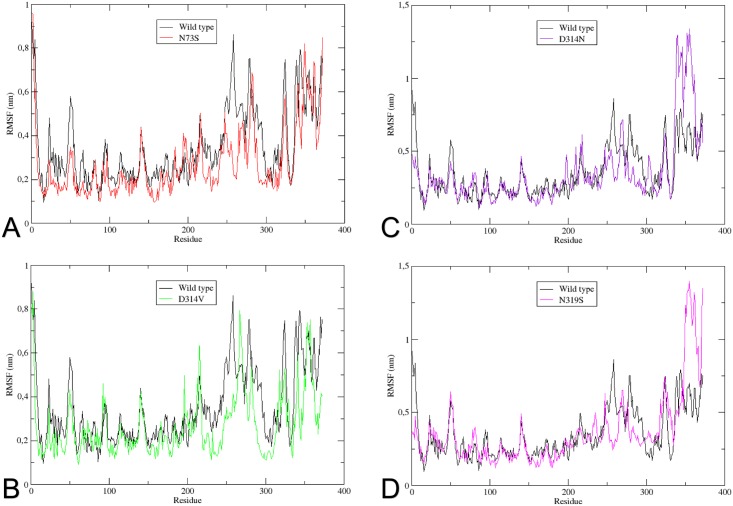
RMSF for each residue of hnRNPA1. The Root-mean-square Fluctuation for each residue of hnRNPA1 is shown. (A) The wild type is represented in black, and mutant N73S in red. (B) The wild type is represented in black, and mutant D314V in green. (C) The wild type is represented in black, and mutant D314N in purple. (D) The wild type is represented in black, and mutant N319S in pink.

The RMSF analysis can be used as a tool to describe local flexibility differences among residues throughout the MD simulation [62]. According to Fig 9, the wild type protein shows an overall higher degree of flexibility when compared to the mutants. A significant difference in RMSF value is seen on residue 258. The wild type protein shows a fluctuation of 0.85nm at Pro258, while the fluctuation at the same position on mutants N73S, D314V, D314N and N319S are 0.2nm, 0.4nm, 0.35nm and 0.3nm respectively, indicating flexibility loss. Mutant N319S shows a significant increase in the RMSF value on the final residues of the protein. Residues 350–372 show fluctuation values ranging from 0.7nm to 1.4nm in this mutant, while in the wild type, these residues fluctuation values range from 0.4nm to 0.75nm. A similar difference in RMSF is shown in the D314N mutant between residues 340 and 360, where the fluctuation values range from 0.7nm to 1.3nm in the mutant, whereas in the wild type it ranges from 0.5nm to 0.7nm. These results suggest that all mutations affect the flexibility of hnRNPA1, particularly the C-terminal end. This general tendency of flexibility loss in mutants may affect protein function, especially regarding the ability of hnRNPA1 to interact with other proteins.

**Fig 10:**
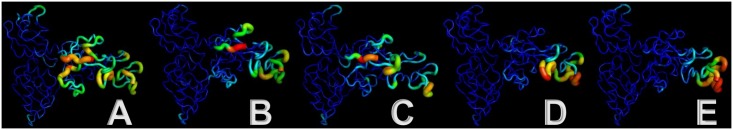
Representation of hnRNPA1 colored according to B-factor. Warm colors indicate high B-factor values, whereas cold colors indicate low B-factor values. (A) Wild type protein. (B) Mutant N73S. (C) Mutant D314V. (D) Mutant D314N. (E) Mutant N319S.

Changes in flexibility can also be evaluated through the analysis of the B-factor, or temperature factor (Fig 10). The B-factor indicates the inherent thermal mobility of protein atoms [64]. The B-factor values achieved after MD simulations were plotted onto the surface of the protein for better visualization. As shown in Fig 10A, the C-terminal end of the wild type protein appears to be a flexible region, with high B-factor values, whereas all the mutants (Fig 10B–10E) exhibit flexibility loss in this region.

### Conservation Analysis

Consurf is a bioinformatics tool that estimates the evolutionary conservation of amino acids in a protein and projects the scores on their molecular surface, using a coloring scheme. The scores are based on the phylogenetic relations between the protein and homologous sequences. Important amino acids are usually strongly conserved throughout evolution, and therefore the level of conservation of an amino acid can indicate the relevance of each amino acid for the protein’s structure or function [47,48,65].

**Fig 11:**
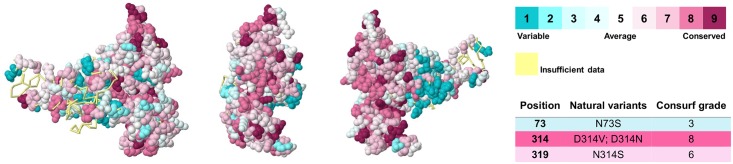
Conservation analysis of each hnRNPA1 amino acid. HnRNPA1 represented as a space-filling model with the conservation grades color-coded onto each amino acid, viewed from three different angles. As the color-coding bar shows, bordeaux indicates highly conserved amino acids, while turquoise indicates variable positions. Amino acids colored in yellow did not receive a conservation score due to insufficiency of data. According to Consurf analysis, position 73 is variable, position 314 is highly conserved and position 319 is conserved.

The final tridimensional model of hnRNPA1 was submitted to the Consurf server and the results are shown in Fig 11. Highly conserved positions are colored bordeaux, intermediately conserved positions are colored white and variable positions are colored turquoise. Consurf rated position 73 as variable, position 319 as intermediately conserved and position 314 as highly conserved. Interestingly, even though the mutation N73S occurs within one of the RNA recognition motifs of hnRNPA1, it was classified as a variable position and considered neutral by all nsSNP prediction algorithms. On the other hand, mutations D314N and N319S take place in conserved positions, which might explain the relation between these mutations and ALS20 development.

## Conclusions

We have successfully modeled the complete tridimensional structure of hnRNPA1, which was proved to be a high-quality model according to PROCHECK, ERRAT, Qmean, ProSA, Rampage and Verify 3D. Mutations D314V, D314N and N319S showed to be deleterious, while mutation N73S was classified as neutral according to the nsSNP predictor algorithms used in this work. According to SNP Effect prediction, mutations D314V and D314N tend to increase amyloid propensity. The nsSNP analysis showed that it is necessary to use more than one prediction algorithm to determine the potential effects of mutations. With the great amount of biological data available today, the fast results obtained with bioinformatics are necessary, however, it is important to note that the *in silico* analysis is not conclusive without the support and validation from the experimental methods. As of today, bioinformatics serve as an ally of the experimental method by making predictions and filtering which results should be thoroughly examined with experiments. The MD simulation results suggest that all mutations increase protein stability when compared to the wild type protein. The Rg results show that, except for mutant N73S, all other mutations significantly affect protein size and level of compaction: mutant D314V exhibits an increase in the level of compaction, whereas mutants N319S and D314N show a decrease in the level of compaction when compared to the wild type protein. These results can be supported by the SASA analysis, which showed that mutant N73S did not affect the accessible surface of the protein, while mutants D314N and N319S showed an increase in the solvent accessible surface of the protein when compared to the wild type. The RMSF analysis along with the B-factor analysis indicated that the flexibility level of the residues in the C-terminal end of hnRNPA1 is affected by all mutations, which may affect the protein ability to interact with other proteins. According to Consurf, residue 73 was classified as variable, even though it is located within one of the RNA recognition motifs of hnRNPA1. Residue 319 was classified as intermediately conserved and residue 314 as highly conserved, which might explain the relation between mutations D314N and N319S with ALS20 development.

## Supporting Information

S1 Fig Backbone RMSD of mutant D314N throughout a 100ns MD simulation. To confirm that mutant D314N stayed stable with an RMSD of 0.6nm, we carried a 100ns MD simulation. The mutant seemed to stabilize at 12ns with an RMSD of 0.5nm, but an increase is noticeable at 40ns, when the backbone RMSD achieves 0.6nm. As expected, this value remained constant until the end of the trajectory without major increases or decreases, indicating that the trajectory stabilized with an RMSD of 0.6nm.
(TIF)
