# Supplementary figures and images for: Amyotrophic Lateral Sclerosis Type 20 - In Silico Analysis and Molecular Dynamics Simulation of hnRNPA1
Source: PLoS One. 2016 Jul 14;11(7):e0158939. doi: 10.1371/journal.pone.0158939 (PMC4945010; doi:10.1371/journal.pone.0158939)

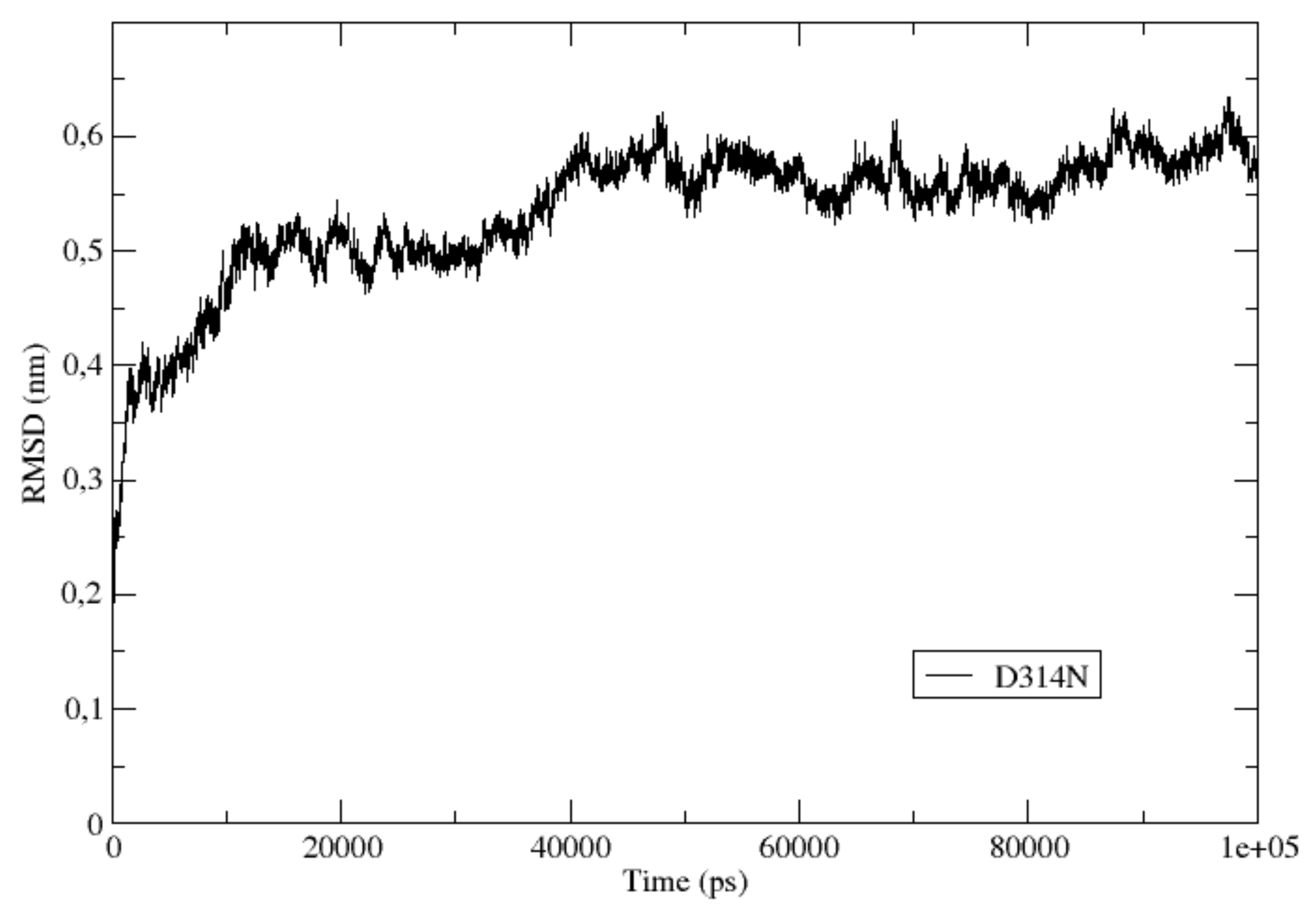

Supplement: S1 Fig — To confirm that mutant D314N stayed stable with an RMSD of 0.6nm, we carried a 100ns MD simulation. The mutant seemed to stabilize at 12ns with an RMSD of 0.5nm, but an increase is noticeable at 40ns, when the backbone RMSD achieves 0.6nm. As expected, this value remained constant until the end of the trajectory without major increases or decreases, indicating that the trajectory stabilized with an RMSD of 0.6nm. (TIF) [file pone.0158939.s001.tif]
